# Simple but Significant Modifications of High-Flow Nasal Cannula

**DOI:** 10.7759/cureus.22641

**Published:** 2022-02-26

**Authors:** Ashutosh K Singh, Manpreet Kaur, Nishant Patel, Richa Aggarwal, Anjan Trikha

**Affiliations:** 1 Department of Anesthesiology, Pain Medicine and Critical Care, All India Institute of Medical Sciences, New Delhi, IND; 2 Department of Critical and Intensive Care, Jai Prakash Narayan (JPN) Apex Trauma Centre, All India Institute of Medical Sciences, New Delhi, IND

**Keywords:** covid-19, high-flow nasal canula, hfnc, modification, innovation

## Abstract

High-flow nasal cannula (HFNC) is one of the most commonly used devices for oxygen therapy during the coronavirus disease 2019 (COVID-19) pandemic in different hospital settings. Multiple alternative options include non-invasive and invasive ventilation. But non-invasive ventilation is very uncomfortable for patients, and weaning from invasive ventilation in a patient with lung pathology is challenging. Hence, HFNC has come up as a safe alternative that averts invasive ventilation. However, its widespread application is difficult in patients with nasal deformities. We discuss two patients, one with caudal dislocation of the nasal septum with a crooked nose and the other patient with septal hypertrophy. In both cases, invasive ventilation was deferred, and target oxygen saturation was achieved after a simple dispositive modification.

## Introduction

High-flow nasal cannula (HFNC) is one of the most commonly used devices in coronavirus disease 2019 (COVID-19) patients with acute respiratory failure owing to the provision of high oxygen concentration, comfort, facilitation of secretion removal, patient compliance, and ease of proning. It delivers humidified oxygen up to a flow rate of 60 L/min and provides a constant FiO_2_ (up to 1.0), positive end-expiratory pressure (PEEP) and reduces dead space and work of breathing [1]. It can reduce the requirement for mechanical ventilation (invasive or non-invasive) and expedite early discharge from the intensive care unit (ICU) [1]. However, it carries the risk of mouth dryness, nasal trauma, pneumothorax, and pneumomediastinum and can delay intubation [2]. Though there are no absolute contraindications for HFNC application, device-related or patient-related factors should be considered before its application [3]. We discuss two cases in which both patients had a nasal deformity with the nasal obstruction which caused either difficulty in placing nasal prongs or obstruction of the nasal prong of the HFNC.

## Case presentation

Case 1

**Figure 1:**
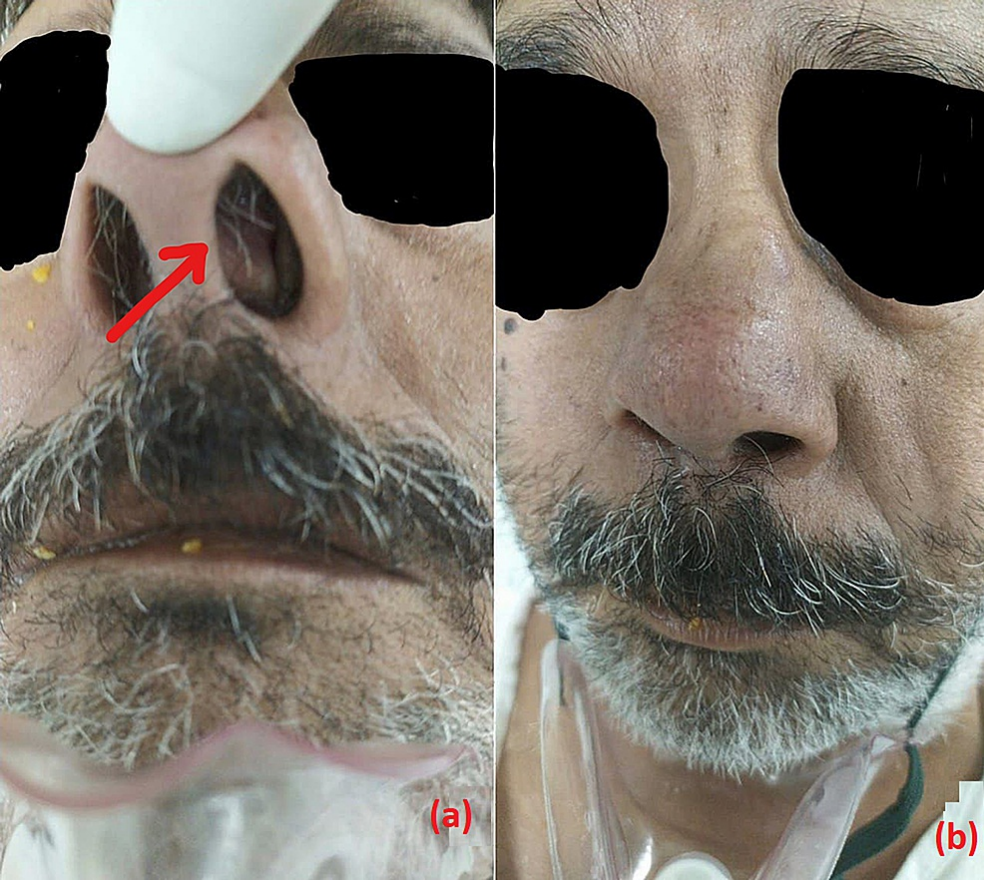
Caudal portion of the nasal septum was dislocated causing severe nasal obstruction in the left nostril (a) and the crooked nose (b).

A 52-year-old male (75 kg) without any comorbidities was admitted to the critical care unit with a positive severe acute respiratory syndrome coronavirus 2 (SARS-CoV-2) reverse transcription-polymerase chain reaction (RT-PCR) result. His room air (SpO_2_) was 64% and 90% with a non-rebreathing mask (NRBM). His CT-severity score was 18/25. Therefore, for better titration and control of FiO_2_, we placed the patient on HFNC. We observed that the HFNC was unable to achieve the optimum SpO_2_ because the base of the nasal prongs was obstructed due to displacement. After local examination and ENT consultation, we found that the caudal portion of the nasal septum was dislocated that causing severe nasal obstruction in the left nostril and the crooked nose (Figures 1a, 1b).

The nasal prongs of the HFNC did not fit because of left nostril obstruction; therefore, displacement and obstruction at the base of the nasal cannula were inevitable. Therefore, we cut half of the nasal cannula for the left nostril so that it was easily fitted and undisplaced without any obstruction after application. Therefore, for better titration of FiO_2_, we applied HFNC and achieved the target SpO_2_.

Case 2

**Figure 2:**
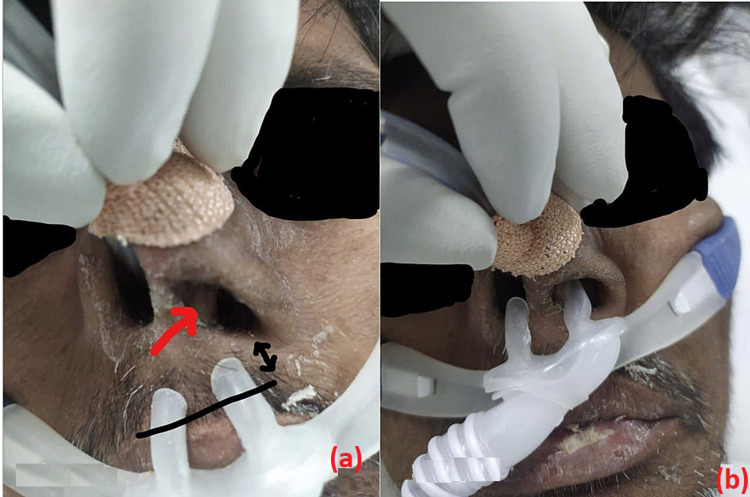
Septal hypertrophy protruded into the left nasal cavity (a) and HFNC nasal cannula impinging over nasal septum (b). HFNC: high-flow nasal cannula

A 41-year-old male with diagnosed alcohol-related decompensated chronic liver disease with portal hypertension, moderate ascites, and hepatic encephalopathy grade 2 was admitted with SARS-CoV-2 in ICU. He had deranged prothrombin time/international normalized ratio (PT/INR) without any hematemesis or melena. The patient’s serum creatinine was 5.4 mg/dL. Due to the high oxygen requirement, we attempted to administer HFNC for maintaining oxygenation. But we observed there was septal hypertrophy and it protruded in the left nasal cavity. Ryles tube was in-situ in the right nasal cavity. So nasal prong was a bit difficult to insert and quite uncomfortable for the patient (Figures 2a, 2b). So we cut one-third part of the left nasal cannula and applied lignocaine jelly over nasal prongs, which made the prongs tolerable and target oxygen saturation was achieved.

## Discussion

The initial management of hypoxic COVID-19 patients is to provide oxygen via different interfaces with targeted SpO_2_ (92-96%). The interface could be a simple facemask, non-rebreathing mask, HFNC, or mechanical ventilation. Patients should be continuously monitored or assessed for the requirement of invasive ventilation so that the patient can be put on the ventilator before he worsens [3]. Proning helps to reduce shunt fraction and improve oxygenation during worsening of dyspnea [4,5]. We followed the same guidelines during the management of hypoxic COVID-19 patients.

HFNC has benefitted innumerable patients suffering from type 1 respiratory failure. It has prevented or reduced the need for ventilation in hypoxemic patients. HFNC application is the last resort to combat hypoxia before mechanical ventilation so that many patients who are prone responsive are saved from intubation. Despres et al. and Xu et al. observed that self proning with HFNC could be a good alternative to reduce shunt fraction and improve oxygenation (PaO_2_/FiO_2_ ratio) in COVID-19 patients [6,7]. It helped to avoid intubation in index series. Both cases were prone responsive and their respiratory drive was not that much, so we tried HFNC with slight modification and proning with NRBM we found good results in oxygenation.

Kumar et al. found improvement in oxygenation with dual oxygen therapy in COVID-19 patients [8]. They applied non-invasive ventilation (NIV) over nasal cannula (oxygen flow at 10 L/min) to decrease rebreathing and dead space and found good results in their cases. We could not apply this technique here because both of our cases were non-compliant with NIV. 

Creativity and clinical judgment on the part of the treating team are paramount when encountered with challenges in delivering novel therapy, such as high-flow nasal cannula, especially in pandemic times. Of course, the safety of the improvised techniques can also be shared among the medical community for larger benefits.

Anatomy of the nasal airway is such that the majority of the patients have deviated nasal septum and can be clinically benefitted if they cannot tolerate HFNC due to nasal impingement. The novel alteration in both the patients by cutting the nasal prong to half to one-third prevented intubation. We could explore the utility of HFNC in prone responsive, non-invasive ventilation noncompliant patients. Such case reports are important so that the manufacturers can make certain adjustments with these oxygen supply devices. An attempt to make these nasal prongs more size adjustable is a reasonable option that can be explored as cutting the prongs too much will take off the benefit of generating adequate positive end-expiratory pressure (PEEP). We make a strong recommendation that this simple dispositive modification can benefit other nasal deformities.

## Conclusions

Hence, HFNC not only provides positive pressure ventilation but also averts intubation and is a reasonable choice in hypoxemic patients. Nasal anomalies may prevent easy use of HFNC but such problems can be easily tided over by simple modification like a slight cut at the nasal prong part, thereby preventing invasive ventilation. It is not only beneficial to the individual patient needing HFNC but also opens up the scope for using ventilators in those patients who really need them but don't get it due to the paucity of ventilators.
